# Systematic analysis of human kinase genes: a large number of genes and alternative splicing events result in functional and structural diversity

**DOI:** 10.1186/1471-2105-6-S4-S20

**Published:** 2005-12-01

**Authors:** Luciano Milanesi, Mauro Petrillo, Leandra Sepe, Angelo Boccia, Nunzio D'Agostino, Myriam Passamano, Salvatore Di Nardo, Gianluca Tasco, Rita Casadio, Giovanni Paolella

**Affiliations:** 1 Biomedical Technologies Institute (ITB), National Research Council, Milano, Italy; 2 CILEA, Segrate, Italy; 3 CEINGE Biotecnologie Avanzate, Napoli, Italy; 4 Dipartimento di Biochimica e Biotecnologie Mediche, Universita' di Napoli Federico II, Italy; 5 Department of Biology, University of Bologna, Italy; 6 Dipartimento SAVA, Universita' del Molise, Italy

## Abstract

**Background:**

Protein kinases are a well defined family of proteins, characterized by the presence of a common kinase catalytic domain and playing a significant role in many important cellular processes, such as proliferation, maintenance of cell shape, apoptosys. In many members of the family, additional non-kinase domains contribute further specialization, resulting in subcellular localization, protein binding and regulation of activity, among others. About 500 genes encode members of the kinase family in the human genome, and although many of them represent well known genes, a larger number of genes code for proteins of more recent identification, or for unknown proteins identified as kinase only after computational studies.

**Results:**

A systematic in silico study performed on the human genome, led to the identification of 5 genes, on chromosome 1, 11, 13, 15 and 16 respectively, and 1 pseudogene on chromosome X; some of these genes are reported as kinases from NCBI but are absent in other databases, such as KinBase. Comparative analysis of 483 gene regions and subsequent computational analysis, aimed at identifying unannotated exons, indicates that a large number of kinase may code for alternately spliced forms or be incorrectly annotated. An InterProScan automated analysis was perfomed to study domain distribution and combination in the various families. At the same time, other structural features were also added to the annotation process, including the putative presence of transmembrane alpha helices, and the cystein propensity to participate into a disulfide bridge.

**Conclusion:**

The predicted human kinome was extended by identifiying both additional genes and potential splice variants, resulting in a varied panorama where functionality may be searched at the gene and protein level. Structural analysis of kinase proteins domains as defined in multiple sources together with transmembrane alpha helices and signal peptide prediction provides hints to function assignment. The results of the human kinome analysis are collected in the KinWeb database, available for browsing and searching over the internet, where all results from the comparative analysis and the gene structure annotation are made available, alongside the domain information. Kinases may be searched by domain combinations and the relative genes may be viewed in a graphic browser at various level of magnification up to gene organization on the full chromosome set.

## Background

Eukaryotic protein kinases (ePKs) are important players in virtually every signalling pathways involved in normal development and disease: the transduction, amplification and integration of many intracellular and intercellular processes need a specific regulation made, often, by protein phosphorylation [1]. Most ePKs belong to a single superfamily, characterized by a contiguous stretch of approximately 250 aminoacids that constitutes the catalytic domain (ePK domain) [2]. A much smaller number of protein kinases do not share this catalytic domain with other kinases, and are often collectively called atypical kinases. The availability of complete sequences for human and vertebrate genomes stimulated the computational search of the whole sequence, in order to identify additional unknown protein kinases: Swissprot, Uniprot, ENSEMBL [3-5] and other commonly used databases all annotate different numbers of kinase proteins or genes. Manning et al [6], in a systematic attempt to establish the full set of human kinases (kinome), identified 478 ePKs and 106 kinase pseudogenes in human genome; apart from 40 atypical protein kinases lacking sequence similarity in the ePK domain. This study was more recently extended to cover additional species [7]. In many cases the ability of protein kinases to regulate biological events depends on the presence, along with the kinase domain, of other functional domains involved in regulation, interactions with other protein partners or subcellular localization [8]. These non-catalytic domains extend the already wide diversification of these proteins, and, at the same time, offer a contribute to explain the high degree of this functional diversification, suggesting alternative targets for structural and functional analysis.

In this article we try to expand the kinase superfamily by identifying novel protein kinases, either encoded in previously unidentified genes or generated via alternative processing steps from the known ones, via comparative genomics analysis. We also try to characterize the gene products by protein sequence analysis, by means of different tools of structure/domain prediction, including some machine learning-based methods specifically developed [9,10] to predict transmembrane alpha helices and disulfide bond propensity for cysteine residues. The data are collected in a database, where all the information from the present study may be publicly accessed.

## Results and discussion

### The human kinase gene set

The exact number of protein kinases present in the human genome is still debatable. The attempt of Manning et al. to identify, by a combination of computational and laboratory findings, the full set of human kinases, showed that the number is much less than originally expected, and close to 500; the exact number is still subject to change, following the refinements of the genome sequence, and the introduction of better prediction tools. The INTERPRO ID IPR000719, corresponding to the domain name "Protein kinase" [8], finds 500 human genes in ENSEMBL, 396 in UNIPROT-Swissprot section and 1268 in the full Uniprot; a set of 452 unique entries in ENSEMBL refer to the gene ontology entries for protein kinase activity or more specific definitions. Kinbase, currently the most comprehensive pool of human kinases, lists 518 kinases (478 as typical ePKs and 40 atypical aPKs) together with 106 pseudogenes. While performing the present analysis, Caenepeel et al. [7] published the mouse kinome, identifying 510 mouse genes as orthologs of the 518 human KinBase kinases; as for human kinases, many mouse genes were not mapped or annotated in ENSEMBL. The KinBase collection, the most accurate set available to date, was used as the starting data set for the present work: a PSSM obtained by PSI-BLAST analysis of this set, using a 90 aminoacids input sequence from the catalytic domain, was used to search for kinases in the full human assembly. This workflow, described under "Methods" led to the identification of 5 genes on chromosome 1, 11, 13, 15, 16 respectively and 1 pseudogene on chromosome X. One of them (*PIK4CB*) is annotated as a kinase and four other as predicted genes in NCBI; all are absent from the Kinbase database (Table 1). Moreover, 7 pseudogenes and the '*KIS*' kinase gene presented different chromosomic localization from the KinBase annotation.

### Kinase gene analysis

**Figure 1:**
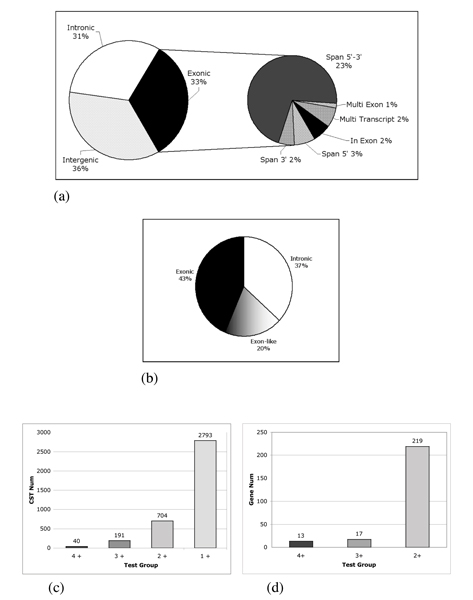
**CST distribution**. (a) Full set of CSTs found in the selected genomic regions (See Methods), grouped according to ENSEMBL gene and exon annotation. Exonic CSTs are indicated as *In exon *when completely included in one exon, *Span 5' *or *Span 3' *when overlapping the 5' or 3' end of one exon, *Span 5'-3' *when CST contains one exon, *Multi exon *or *Multi Transcript *when a larger CST covers more than one exon of one or more transcripts. (b) CSTs contained within the analysed kinase genes: 20% of the CSTs, initially annotated as intronic, are indicated as "exon-like" because positive to one or more of the tests described under methods. (c) Exon-like CSTs within the analysed kinase genes, positive to one, two, three and four of the used test criteria (see methods). (d) Number of kinase genes containing at least one exon-like CST positive to two, three or four tests.

Starting from the new pool of kinase genes, including the 518 from Manning and 5 new ones from our analysis, we proceeded to a comparative analysis of man and mouse orthologous kinase genes, aimed to the identification of small genomic conserved sequence tags (CSTs). As functional sequences tend to evolve at a slower rate than neutral sequences, information about conserved regions in these genes, obtained from alignment of DNA sequences from different species, such as human and mouse, provides an useful opportunity to amplify the knowledge about coding sequences, alternatively spliced exons or about other regulatory elements. The analysis was performed according to a tool, previously developed in our lab for identification of CSTs in disease genes [11]. Briefly, given a list of target genes, human genomic regions spanning the selected transcripts are identified and compared to their mouse counterparts as defined according to ENSEMBL orthologous definitions; when the target genes are very close, they are included in the same genomic region. Stretches longer than 100 bp and sharing a minimum of 70% identity, are selected as CSTs and assigned to the nearest gene. As ENSEMBL gene codes were not available for all human kinases, gene codes for the kinase gene set were determined according to their gene name or contig position and manually verified. From the initial set, 483 could be analyzed: sequences from man and mouse, corresponding to genomic regions spanning the selected kinase transcripts, were extracted from ENSEMBL, masked for repeats and compared for the identification of CSTs. The comparison led to the identification of about 35000 human (and mouse) CSTs, which together cover about 8% of the selected human regions. Kinase CSTs are mostly unique and half of them correspond to the expected exons for genes contained in the analyzed regions. A summary of the CSTs grouped by gene type is reported in Fig. 1a. The CST collection underwent several annotation steps involving:

• basic features concerning sequence content and similarity with orthologous counterpart, such as GC%; number of gaps, length, sequence identity, polarity.;

• genomic localization, according to ENSEMBL data and concerning chromosomal coordinates, relationship with the target and the closest gene (i.e. distances from transcription and coding start, transcription and coding end, according to gene orientation), sequence gene type (i.e. intergenic, intronic, exonic), number of known SNPs;

• predicted functional features, concerning the identification of motifs and putative signals, such as transcription factor binding sites, exonic splicing enhancers (ESE), RNA secondary structures, palindromes and tandem repeats.

Many CTSs reported in Fig. 1a as intergenic or intronic (i.e. non-exonic), could of course represent additional, previously unidentified exons, either constitutive or alternatively spliced, and their identification would lead to further expansion of the available kinome. Several computational tests were therefore directed to determine the coding potential, including identification of the long open reading frames, calculation of codon frequency and periodicity, statistics on synonymous codon usage, coincidence with exons and suboptimal exons of genes predicted by running GENSCAN on the selected human genomic regions. In addition human and mouse EST collections were scanned by BLAST for similarity to human and mouse CSTs, and different genomic annotations were compared to highlight differences between human and mouse annotations, such as human CSTs annotated as intergenic or intronic with mouse counterpart annotated as exonic. The last four tests, i.e. coincidence with GENSCAN exons, matches with human or mouse EST and annotation as exons in mouse are, alone or in combination, particularly convenient criteria to assess the exon potential: about a third of the CSTs initially marked as intronic or intergenic were positive to at least one of the described criteria and should be considered as "exon-like" (Fig. 1b). Concomitant positivity to more than one criteria is often observed and allows to rank sequences in classes of higher potential to be unannotated constitutive or alternatively spliced exons (Fig. 1c). The number of kinase genes containing such CSTs is large: results are likely to represent unannotated constitutive exons or to code for alternatively spliced isoforms: about half of the genes contain at least one region positive to two or more of the four criteria mentioned above (Fig. 1d).

**Figure 2:**
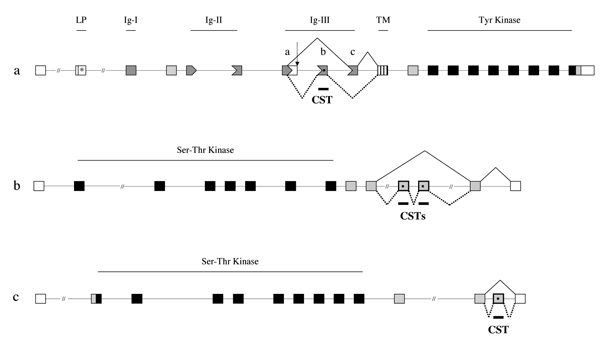
**Some kinase genes containing extra exons. **(a) *FGFR1 *gene. (b) *CDKL2 *gene. (c) *VRK1 *gene. Putative new exons derived from CST analysis are reported are marked by a dot. Splicing patterns are indicated by continuous (known) and dashed (predicted) lines. Exons encoding kinase, transmembrane and Ig-like domains are indicated; white boxes represent untranslated RNA sequence, a star marks the leader peptide of FGFR1.

Manual analysis of some exon-like CSTs, identified with the above described procedure, revealed potential kinase isoforms that could be validated against information currently available in literature. The human gene encoding fibroblast growth factor receptor 1 (*FGFR1*) includes an unannotated exon which, if inserted into the mRNA, produces a *FGFR1*-IIIb form, not currently annotated in the used databases. This exon is consistent with the common gene organization seen both in man and mouse in the genes coding for other FGFR isoforms, which underlies the mechanisms for generating receptors differing in their third immunoglobulin domain: three different exons (IIIa, IIIb and IIIc) encode possible alternatives within the third Ig-like domain at the N-terminal end of the molecule (Fig. 2a). A literature search provided experimental evidence supporting the existence of *FGFR1*-IIIa and IIIc transcript variants both in human and mouse, and a cDNA containing the IIIb exon in mouse. A recombinat protein containing the human IIIb sequence is active; RNAse protection and PCR experiments confirm the existence of the m-RNA encoding the *FGFR1*-IIIb in mouse and human brain [12-14].

Two CSTs identified in *CDKL2 *(Cycline-dependent kinase-like 2), a member of the cdc2-related serine/threonine kinase subfamily, contain putative exons inserted into the 3' end of the coding region (fig. 2b). The generated protein differs from *CDKL2 *in its C-terminal domain by increasing the length of a random coil region located at the carboxy-end of the protein, a region in which previous analysis of mouse cDNA clones already revealed multiple variants generated by alternative splicing events. These exons are not described in the numerous species where the protein has been reported, with the only exception of rabbit, where a similar sequence from deep cerebellar nuclei is described as the only form available [15]. Despite their similarity with cdc2, most of the members of this family show roles other than cell cycle regulation and are expressed in terminally differentiated cells of the nervous system. Consistently, a human EST ending in a poliA+ tail confirms its inclusion in human brain RNA and provides evidence for a brain specific form of the protein.

Similarly, *VRK1 *(vaccinia-related kinase 1) shows an extra exon contained within CST, which is suggestive of an alternative splicing event localized at the 3' end of the *VRK1 *gene, affecting the low complexity domain at the C-terminus of the protein (Fig. 2c). The protein, identified from a new group of human serine/threonine kinases, known to prevent p53 ubiquitinization via phosphorylation in thr-18 [16,17], only has one isoform according to the SWISSPROT protein database, while five alternative products are reported as predicted for the murine one.

### Structure-function relationship in kinase proteins

**Figure 3:**
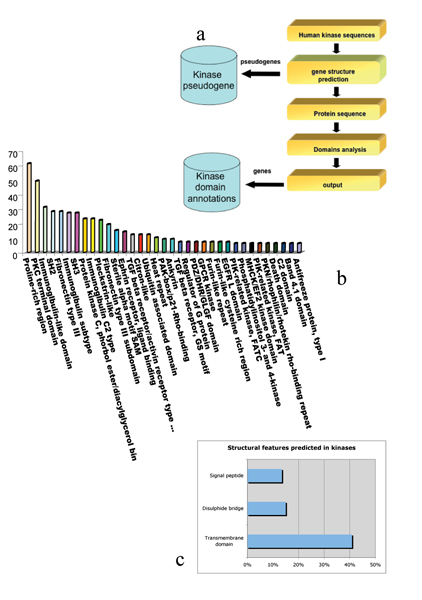
**Comprehensive analysis of protein kinase genes. **(a)The pipeline used for the annotation of the kinase genes is represented in this schema, information deriving from the gene annotations are recorded into the relational database KinWeb. (b) Count of domains identified within the kinase gene pool.(c)Prediction of structure features in kinase protein sequences.

The human kinase genes, in addition to the catalytic domain, contain several other domains involved in various regulation processes. In order to evaluate the domain distribution in all human kinases, an automated procedure was developed based on an INTERPRO-Scan analysis of the protein kinase sequences. The procedure led to the identification of 20 families, 91 different domains, 12 repeats, 1 binding site (Fig. 3a). The domains found are reported in Fig. 3b, sorted by frequency; domains appearing less than 6 times are not reported in figure. A majority of the human protein kinases contains at least one domain other than the catalytic kinase domain. Many domains are useful to function assignment: 60 kinases present domains that interact with nucleic acids, 53 present domains linked to lipid signalling, 37 kinases include domains related to GTPase signalling, 12 kinases present domains involved in calcium signalling, 8 kinases present domains able to target the protein to the cytoskeleton. Some domains are present in multiple copies: 2 to 8 Fibronectin type III domains are present in *TIE*, *AXL*, *ROS*, *InsR *and related kinases, located before the catalytic domain. Src Homology 2 (SH2) and Src Homology 3 (SH3) are adaptor domains involved in the recruitment of proteins to their specific target and are frequently found in kinases of the TK family: 19 kinases contain both domains, while 10 kinases contain the sole SH2 domain and 8 the sole SH3 domain. In kinases such as *TEC*, *SRC*, *CSK *and *ABL*, that contain both the SH3 and SH2 domains, the peptide that connects SH2 and catalytic domain tends to maintain similar lengths, on average 20 amino-acids long. The large number and diversity of non-kinase domains, contained within protein kinases, is directly related to the high degree of functional diversification, which greatly depends on their ability to interact with a large number of other cellular proteins, mainly via additional subunits or domains. The concomitant presence of additional, non-catalytic, domains may thus lead to the understanding of possible interacting partners and intracellular pathways recruited, unltimately hinting to a specific cell function.

Further information in our data mining system comes from an exhaustive prediction of transmembrane domains and other structural features. This information allows to better understand the connection between structure and function of known proteins, but also permits to express hypothesis about the role and the subcellular localization of novel proteins. Filtering of the kinase sequence set with machine-learning based methods, specifically suited to predict signal peptides, transmembrane protein domains of the alpha helical type and propensity of cysteine residues to form disulfide bridges, allowed the annotation on predictive basis of these characteristics. The results are shown in Fig. 3c. We found that 13.5% of the kinase sequences are endowed with signal peptides, suggesting that these proteins may be secreted via the SEC-dependent secretory pathway; 40.9% are endowed with at least one transmembrane domain different from the signal peptide, a number substantially higher than the 18.4% annotated as containing a Tm domain in the Swissprot database; 15% of the kinases are endowed with at least one disulfide bridge.

### KinWeb database

**Figure 4:**
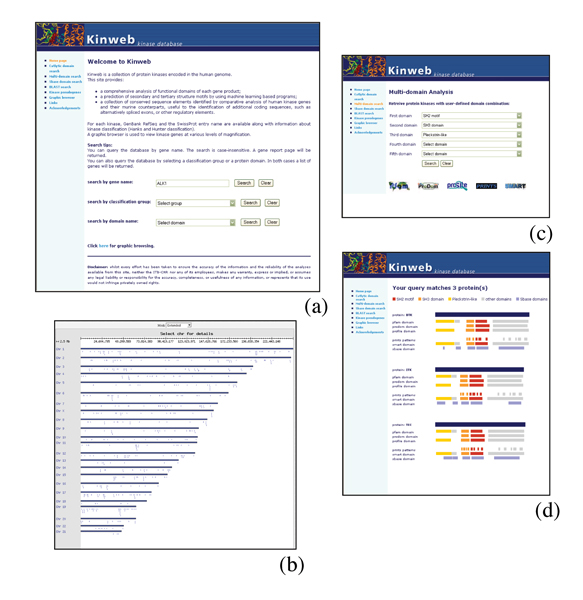
**The KinWeb database. **The interface provides access to the data in various ways: (a) The KinWeb home page. A query form allows to search by gene name, classification group or domain. (b) The genome browser, allowing view and selection of the genomic regions where kinase genes are located. (c) The multi-domain search page. Here kinases can be searched according to a combination of domains. (d) Example of results from multi-domain search in (c). For each protein, all domains predicted by InterProScan are graphically represented.

All the results produced by the analysis have been integrated with the information about kinase genes derived from public databanks into a new database, KinWeb, which is available as a public access site at the following addresses:  and . The human kinome may be accessed through a graphic genome browser and investigated at the genomic level, starting from kinase gene locations, and, progressively adding detail, at the level of gene structure and corresponding CSTs. Kinase genes may be searched on the basis of structural features, such as domain combinations, and various annotations, i.e. gene name or kinase group (Fig. 4). It is also possible to use BLAST for similarity searches between a given sequence and kinase proteins or cDNAs. A sequence may also be compared through HMMER with the full set of available catalitic domains.

The information stored for each kinase gene consists of annotations automatically extracted from public databanks or literature such as:

• alternative names as defined in the HUGO database;

• family classification according to Manning;

• transcript variants and genomic contig names and coordinates from RefSeq;

• functional annotations from Gene-Entrez;

• information about transcripts, exons and genomic coordinates from Ensembl;

• direct links to RefSeq, Gene-Entrez, OMIM, Ensembl and SwissProt databases, together with the Kinbase protein and mRNA sequences.

These annotations are stored alongside the results from the present analysis on kinase genes and proteins. The available data include:

• type and position of detected domains;

• predictions for secondary structure, transmembrane domains and cystein propensity to form disulfur bridges;

• mouse orthologous kinase genes;

• CSTs common to human and mouse;

• all CST annotations.

**Figure 5:**
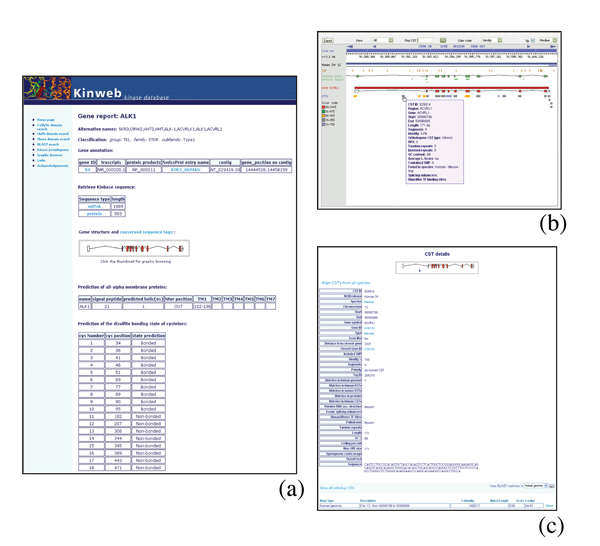
**Annotations available for each kinase gene. **(a) The "Gene Report" page shows gene annotations extracted from public databases, the results of predictions for transmembrane domains and Cys disulfide bonding state and links the identified CSTs to the graphical representation of the gene. (b) Graphical representation of the gene and related transcripts and CSTs. Details are shown for the CTS under the mouse pointer. CSTs are color-coded based on degree of conservation. (c) The CST details page, where full details of the selected CST are shown, including the results of BLAST searches.

The CST elements and their complete annotations are associated with the corresponding gene, but may also be seen, with the help of a graphic browser, in their chromosomal context and in relation to the exons of the gene transcripts; color code is used to label CSTs according to the various annotations, including the number and type of BLAST matches found (Fig. 5). A link leads from each human kinase gene to the orthologous mouse gene, where information on structure and CSTs, also stored within the database and accessible through the graphic browser, are available. Altoghether the data provide an exaustive analysis of various aspects of gene and protein features for each kinase, integrating data from literature and other DBs with information about gene organization, sequence conservation and protein structural predictions, obtained within the present analysis.

## Conclusion

The predicted human kinome was extended by identifying kinase genes through a custom built pipeline and by identifying a large number of non-exonic, apparently non-coding, highly conserved sequences through comparative analysis. Some of these conserved sequences were annotated as exon-like, and may be responsible for additional protein variability through alternative processing; others may play different roles, for example contribute to regulation of gene expression. Domain analysis and prediction of structural features provide further information, resulting in a varied panorama where functionality may be searched at the gene or protein level. All results from the comparative analysis and the gene structure annotation are made available alongside the domain information in the KinWeb database, made available for browsing and searching over the internet and where it is possible to search for kinases by domain combinations and to visualize the relative genes, including annotation of conserved sequences. A graphic browser is used to view kinase genes at various levels of magnification, from single exons up to gene organization on the full chromosome set.

## Methods

### Kinase gene identification

A contiguous stretch of approximately 90 aminoacids, containing the well known "DxxxxN, DFG, APE, DxxxxG" motif, was extracted from an arbitrarly choosen kinase, ABL, and used as input to a three-iteration PSI-BLAST search of a query database containing the whole kinase dataset identified by Manning and coworkers [6]. The resulting Position Specific Score Matrix (PSSM) was used as a query sequence to perform tBLASTn agaist all human chromosome sequences, available from NCBI, release April 14 2003; human sequences had been previously masked to remove sequences coding for kinase genes contained in the starting set. Sequence regions matching the PSSM were extracted and extended 200 kb upstream and downstream for full length gene prediction on the resulting genomic region by GenomeScan . This software allows prediction of genes on the basis of an input protein expected to be similar to the gene product encoded in the DNA sequence. We found such proteins by doing a BLASTX comparison of our sequences to all known proteins.

### Kinase domain identification

For each human hit, all the features (gene name, alternative names, classification) were stored into a table of a relational database, along with protein, mRNA and kinase catalytic domain sequences. Pseudogenes were manually curated and inserted into a distinct relational database. All putative kinases were then analyzed by using the InterProScan for the complete domain annotation. InterProScan [4] is freely available under the GNU licence agreement from the EBI's ftp server . The output generated in XML format, is parsed by a Perl script in order to extract all the annotations and recorded into a MySQL relational database which can be consulted through interfaces written in PHP, and can be visualized with common browsers over the internet.

### CST identification

Human/mouse orthologous regions corresponding to kinase genes were taken from ENSEMBL annotation, when available, or by manual identification based on sequence conservation. The limits of the genomic sequences were set between 20 kb and 250 kb depending on the distance of the closest known gene. Species-specific repeats were masked and BLASTZ was used for comparison. The final set of about 33000 CSTs was finally selected according to the parameters described in the "Results" section.

### CST annotation

Annotation of human and murine CSTs was carried out through a pipeline, formed of several independent modules. The pipeline is based on PHP scripts and includes: classification of CST type according to Ensembl gene definitions, coding capability according to Ensembl exon definitions, GC content, distances from analysed and closest genes and coding regions.

A number of programs, were run on the whole CST set to annotate specific features: equiktandem and palindrome were used to identify direct and inverted repeats; marscan to annotate MAR sites; tcode, syco and getorf to assess coding potential; Genesplicer  to detect splice sites; GENESCAN was used for *ab initio *transcripts and suboptimal exons prediction. equiktandem, palindrome, marscan, tcode, syco and getorf are EMBOSS applications .

### BLAST searches

BLAST was run on all CSTs to annotate matches within the human and mouse genomes: matches showing score higher than 50 or E-value better than 10^-5 ^were kept as annotations in the DB. Similarly results of BLAST runs of all CSTs against human and mouse EST libraries having E-value better than 10^-20^, length and identity higher than 30 and 90 respectively, have been annotated.

### Exon like definition

The CSTs are annotated as exon-like when one or more of the following conditions is verified:

- mouse counterpart is annotated as exonic

- CST matches with GENSCAN exons or suboptimal exons

- CST matches with one or more human EST

- Mouse counterpart matches with one or more rodent EST

### Database construction and web interface

CSTs are fed to an SQL-database. The pipeline is able to manage CST import together with automatic annotation. The WEB interface consists of PHP scripts, which query the database and dynamically generate the result pages. The graphic visualization tool has been developed in PHP and is based on the GD graphics library .

### Signal peptide prediction

The entire set of proteins was filtered with SPEPlip, a neural network-based method for predicting the presence of a signal peptide, trained and tested on a set of experimentally derived signal peptides from eukaryotes and prokaryotes. SPEPlip identifies the presence of sorting signals and predicts their cleavage sites. The accuracy is 97%. It can be accessed through the web page at 

### All-alpha membrane proteins prediction

All-alpha membrane proteins constitute a functionally relevant subset of the whole proteome. Their content ranges from about 10 to 30% of the cell proteins, based on sequence comparison and specific predictive methods. ENSEMBLE is an ensemble of methods, containing a cascade-neural network (NN) and two different hidden Markov models (HMM). It was trained and tested in cross validation on 59 well resolved membrane proteins, available when the method was implemented. ENSEMBLE scores with a per-protein accuracy of 90% for topography and 71% for topology. When tested on a low resolution set of 151 proteins, with no homology with the 59 proteins, the per-protein accuracy of ENSEMBLE is 76% for topography and 68% for topology.

### Disulfide bond prediction

The propensity of disulfide-bonded cysteines has been predicted with a Hidden Neural Network-based method starting from the residue sequence of the protein chain. The method scores as high as 89% and 86% per cysteine residue and per protein, respectively, and in this it is superior to other predictors of the same category.

## Figures and Tables

**Table 1:** Genomic loci identified after the described procedure

**Genes**	**Chr**	**Start**	**End**	**Strand**	**Entrez Gene Name**	**Description**
1	1	148077486	148111797	Minus	*PIK4CB*	phosphatidylinositol 4-kinase catalytic beta polipeptide
2	11	120736301	120739223	Minus	*LOC283155*	similar to bone morphogenetic protein receptor, type IA precursor; activin A receptor, type II-like kinase 3
3	13	51536903	51601228	Minus	*MGC75495*	similar to NEK1 (NimA-related protein kinase 1)
4	15	34878090	34897999	Minus	*LOC161635*	similar to casein kinase I alpha
5	16	2606123	2663452	Minus	*LOC440332*	similar to 3-phosphoinositide dependent protein kinase-1 (hPDK1)
**Pseudogenes**						
1	X	72527746	72529019	Minus	*LOC139201*	mitogen-activated protein kinase kinase 4 pseudogene

## References

[B1] Cohen P (2001). The role of protein phosphorylation in human health and disease. The Sir Hans Krebs Medal Lecture. Eur J Biochem.

[B2] Hanks SK (2003). Genomic analysis of the eukaryotic protein kinase superfamily: a perspective. Genome Biol.

[B3] Bairoch A, Apweiler R, Wu CH, Barker WC, Boeckmann B, Ferro S, Gasteiger E, Huang H, Lopez R, Magrane M, Martin MJ, Natale DA, O'Donovan C, Redaschi N, Yeh LS The Universal Protein Resource (UniProt). Nucleic Acids Res.

[B4] Mulder NJ, Apweiler R, Attwood TK, Bairoch A, Bateman A, Binns D, Bradley P, Bork P, Bucher P, Cerutti L, Copley R, Courcelle E, Das U, Durbin R, Fleischmann W, Gough J, Haft D, Harte N, Hulo N, Kahn D, Kanapin A, Krestyaninova M, Lonsdale D, Lopez R, Letunic I, Madera M, Maslen J, McDowall J, Mitchell A, Nikolskaya AN, Orchard S, Pagni M, Ponting CP, Quevillon E, Selengut J, Sigrist CJ, Silventoinen V, Studholme DJ, Vaughan R, Wu CH (2005). InterPro, progress and status in 2005. Nucleic Acids Res.

[B5] Hubbard T, Andrews D, Caccamo M, Cameron G, Chen Y, Clamp M, Clarke L, Coates G, Cox T, Cunningham F, Curwen V, Cutts T, Down T, Durbin R, Fernandez-Suarez XM, Gilbert J, Hammond M, Herrero J, Hotz H, Howe K, Iyer V, Jekosch K, Kahari A, Kasprzyk A, Keefe D, Keenan S, Kokocinsci F, London D, Longden I, McVicker G, Melsopp C, Meidl P, Potter S, Proctor G, Rae M, Rios D, Schuster M, Searle S, Severin J, Slater G, Smedley D, Smith J, Spooner W, Stabenau A, Stalker J, Storey R, Trevanion S, Ureta-Vidal A, Vogel J, White S, Woodwark C, Birney E Ensembl 2005. Nucleic Acids Res.

[B6] Manning G, Whyte DB, Martinez R, Hunter T, Sudarsanam S (2002). The protein kinase complement of the human genome. Science.

[B7] Caenepeel S, Charydczak G, Sudarsanam S, Hunter T, Manning G (2004). The mouse kinome: Discovery and comparative genomics of all mouse protein kinases. PNAS.

[B8] Krupa A, Srinivasan N (2002). The repertoire of protein kinases encoded in the draft version of the human genome: atypical variations and uncommon domain combinations. Genome Biol.

[B9] Fariselli P, Finocchiaro G, Casadio R SPEPlip: the detection of signal peptide and lipoprotein cleavage sites. Bioinformatics.

[B10] Martelli PL, Fariselli P, Casadio R (2003). An ENSEMBLE machine learning approach for the prediction of all-alpha membrane proteins. Bioinformatics.

[B11] Boccia A, Petrillo M, di Bernardo D, Guffanti A, Mignone F, Confalonieri S, Luzi L, Pesole G, Paolella G, Ballabio A, Banfi S DG-CST (Disease Gene Conserved Sequence Tags), a database of human-mouse conserved elements associated to disease genes. Nucleic Acids Res.

[B12] Johnson DE, Lu J, Chen E, Werner S, Williams LT (1991). The human fibroblast growth factor receptor genes: a common structural arrangment underlies the mechanism for generating receptor forms that differ in their third immunoglobulin domain. Mol Cell Biol.

[B13] Werner S, Duan DS, de Vries C, Peters KG, Johnson DE, Williams LT (1992). Differential splicing in the extracellular region of fibroblast growth factor receptor 1 generates receptor variants with different ligand-binding specificities. Mol Cell Biol.

[B14] S Beer HD, Vindevoghel L, Gait MJ, Revest JM, Duan DR, Mason I, Dickson C, Werner S Fibroblast growth factor (FGF) receptor 1-IIIb is a naturally occurring functional receptor for FGFs that is preferentially expressed in the skin and the brain. J Biol Chem.

[B15] Sassa T, Gomi H, Itohara S (2004). Postnatal expression of Cdkl2 in mouse brain revealed by LacZ inserted into the Cdkl2 locus. Cell Tissue Res.

[B16] Nezu J, Oku A, Jones MH, Shimane M Identification of two novel human putative serine/threonine kinases, VRK1 and VRK2, with structural similarity to vaccinia virus B1R kinase. Genomics.

[B17] Vega FM, Sevilla A, Lazo PA (2004). p53 Stabilization and accumulation induced by human vaccinia-related kinase 1. Mol Cell Biol.

[B18] Burge C, Karlin S (1997). Prediction of complete gene structures in human genomic DNA. J Mol Biol.

